# Genomic Mosaicism Formed by Somatic Variation in the Aging and Diseased Brain

**DOI:** 10.3390/genes12071071

**Published:** 2021-07-14

**Authors:** Isabel Costantino, Juliet Nicodemus, Jerold Chun

**Affiliations:** 1 Translational Neuroscience Initiative, Sanford Burnham Prebys Medical Discovery Institute, La Jolla, CA 92037, USA; icostantino@sbpdiscovery.org (I.C.); jnicodemus@sbpdiscovery.org (J.N.); 2 Neurosciences Graduate Program, School of Medicine, University of California San Diego, La Jolla, CA 92093, USA

**Keywords:** Alzheimer’s disease, amyotrophic lateral sclerosis, aneuploidy, copy number variation, Parkinson’s disease, repeat expansion, retrotransposons, single-nucleotide variation, somatic gene recombination, somatic variation

## Abstract

Over the past 20 years, analyses of single brain cell genomes have revealed that the brain is composed of cells with myriad distinct genomes: the brain is a genomic mosaic, generated by a host of DNA sequence-altering processes that occur somatically and do not affect the germline. As such, these sequence changes are not heritable. Some processes appear to occur during neurogenesis, when cells are mitotic, whereas others may also function in post-mitotic cells. Here, we review multiple forms of DNA sequence alterations that have now been documented: aneuploidies and aneusomies, smaller copy number variations (CNVs), somatic repeat expansions, retrotransposons, genomic cDNAs (gencDNAs) associated with somatic gene recombination (SGR), and single nucleotide variations (SNVs). A catch-all term of DNA content variation (DCV) has also been used to describe the overall phenomenon, which can include multiple forms within a single cell’s genome. A requisite step in the analyses of genomic mosaicism is ongoing technology development, which is also discussed. Genomic mosaicism alters one of the most stable biological molecules, DNA, which may have many repercussions, ranging from normal functions including effects of aging, to creating dysfunction that occurs in neurodegenerative and other brain diseases, most of which show sporadic presentation, unlinked to causal, heritable genes.

## 1. Introduction

Beginning with the writings of Ramón y Cajal around the start of the 20th century [1,2], a striking characteristic of the vertebrate nervous system was identified through documentation of the enormous diversity and complexity of its constituent cells, which increases with age [3,4], wherein no two cells are truly identical. The molecular mechanisms underlying this diversity remain only partially known; however, they must extend beyond morphology to physiological and functional diversity, as epitomized by the complex visual system [5,6,7,8] and single-cell physiologies [9]. Elements of cellular and functional diversity likely underlie most, if not all, of the brain’s activities including those with temporal stability, such as long-term memory [10,11,12]. These properties of the brain have implicated possible diverse changes to molecules involved with stable, biological information storage, particularly affecting DNA.

The notion that genomic DNA sequence changes—distinct from epigenetic changes that do not alter sequence [13]—might underlie the normal complexity of the nervous system emerged in the 1960s during theoretical explanations of generating antibody diversity [14], which initiated discussions and the search for evidence that DNA sequences could vary amongst cells from the same brain. This possibility has now been born-out in many studies that demonstrated genomically mosaic brains are composed of cells with distinct DNA sequences. Critically, these DNA sequence changes occur somatically rather than affecting the germline and are not heritable; thus, the term “genomic” rather than “genetic” [15] mosaicism is used to underscore somatic alterations lacking heritability.

**Figure 1:**
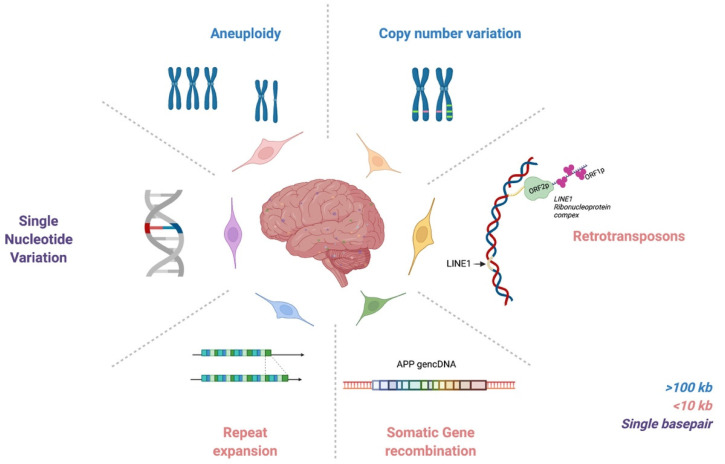
Neural somatic DNA content variation (DCV) encompasses many forms and sizes of somatic changes. Aneuploidies/aneusomies and copy number variations (CNVs) typically reflect large genomic gains/losses that may affect many hundreds of genes. Retrotransposons and somatic gene recombination are reverse transcriptase-mediated insertions of nucleic acids into the genome. Repeat expansions reflect increases in length of instable, small repeating sequences. Single nucleotide variations (SNVs) are the smallest, but most frequent, DNA changes. They encompass changes from one base to another or insertions/deletions of a single base. Together, these somatic changes create genomic mosaicism throughout the human brain.

It is notable that analyses of single-cell genomes have required hand-in-hand technology development, which in part explains the prolonged gap between initial hypothesis generation in the 1960s and actual identification of genomic mosaicism. Twenty years ago, the first evidence for pervasive genomic changes in the brain was reported through chromosomal aneuploidies and aneusomies [16]. In the ensuing years, a range of DNA sequence forms, altered within single cells, has also been reported, from the largest form as aneuploidies and aneusomies, to smaller copy number variants (CNVs), somatic repeat expansion, retrotransposons, genomic cDNAs (gencDNAs), and single nucleotide variations (SNVs) (Figure 1). These elements are reviewed here as they relate to the normal, aging, and neurodegenerative diseased brain. The critical role for technology that is continuously improving is also discussed, along with limitations. Genomic mosaicism appears to change with aging and disease, which has both mechanistic and therapeutic implications.

## 2. DNA Content Variation & Aneuploidy

### 2.1. DNA Content Variation & Aneuploidy in Health and Aging

The first form of neural somatic variation identified was mosaic aneuploidy among mitotic neural progenitor cells [16]. Aneuploidy is defined as the gain and/or loss of chromosomes from the euploid complement. Aneusomy—where a partial chromosomal assessment is made without knowledge of all other chromosomes—was identified in postmitotic neurons of mouse and human using spectral karyotyping and fluorescence in situ hybridization (FISH) [17,18,19,20,21]. The literature tends to be imprecise about the use of the terms “aneuploidy” vs. “aneusomy,” and for the remainder of this review, they will be used synonymously, with the proviso that they in fact have distinct meanings. In the adult brain, FISH studies estimated that ~10% of cells are aneuploid [21,22,23,24,25,26]. Iourov et al. utilized FISH and interphase chromosome-specific multicolor banding to establish a rate of aneuploidy in 12 non-diseased brains. While different chromosomes had comparative levels of aneusomy (0.4–1.2%), mean levels varied widely amongst chromosomes and individuals, with chromosome loss more common than gain [21]. However, ~10% may be an overestimate of aneuploidy. Knouse et al. utilized single cell whole genome sequencing (scWGS) of 89 cells from the frontal lobe of four individuals (48–70 years). Of those, only two cells were aneuploid (Chr22 monosomy and Chr18 trisomy). Therefore, they concluded that the aneuploidy rate in the adult human brain is 2.2% [27]. This rate of aneuploidy has since been corroborated in other scWGS studies of non-diseased brains [28], with the caveat of very limited sample sizes relative to the 170 billion cells of the human brain.

Aneuploidy, along with other substantial somatic variants, such as retrotransposons and copy number variants, can be captured by DNA content variation (DCV) [25,29]. DCV appears widespread within the non-diseased brain. A greater DNA content in cortical neurons as compared to cortical glia, cerebellar cells, and lymphocytes, has been demonstrated using propidium iodide (PI) flow cytometry [29]. Slide based cytometry of multiple cortical regions from healthy individuals revealed that 11.5% of neurons show increased DNA content above diploid level, with no major regional differences within the cortex (non-cortical areas such as the cerebellum were not examined) [30]. The frequency of neurons with high DCV declines with age, indicating that increased DCV might compromise neuronal viability in the aging brain or that high DCV may predispose individuals to aging-related neurodegenerative diseases [30], a possibility supported by increased DCV observed in sporadic Alzheimer’s disease (SAD) [31].

There are multiple mechanisms through which somatic DCV can occur in the brain. DCV and aneuploidy can have a developmental origin, resulting from mitotic failures in development. Alternatively, postmitotic neurons in the adult brain can synthesize excess DNA de novo through dysfunctional neuronal DNA repair [32,33,34]. Many questions about the prevalence and mechanism of formation of DCV and aneuploidy in the normal, aging, and diseased brain remain, requiring further examination through multimodal experimental approaches. A compilation of found somatic variants by disease type and experimental methodology is available in Appendix A.

### 2.2. DNA Content Variation & Aneuploidy in Neurodegenerative Disease

Changes in DCV and aneuploidy have been reported in multiple neurodegenerative diseases, including ataxia-telangiectasia (A-T) and Alzheimer’s disease (AD) [21,25,35,36]. A-T is an autosomal recessive primary immunodeficiency disease caused by mutations in ataxia-telangiectasia mutated kinase (*ATM*), a gene critical in preventing deleterious non-homologous end joining [37]. Patients present in childhood with multiple nervous and immune system disorders, including progressive cerebellar ataxia, oculocutaneous telangiectasia, immunodeficiencies, metabolic diseases, and increased susceptibility to malignancies [38]. Iourov et al. observed a 2–3-fold increase in stochastic aneuploidy across different chromosomes in neurons of the cerebellum and cortex of the A-T brain using FISH [24]. Furthermore, the cerebellum in A-T demonstrated a 5–20-fold increase in DNA double-strand breaks and aneuploidy affecting chromosomes 14, 7, and X. These data, combined with previous research in *ATM* knockout mice demonstrating aneuploidy in 40% of the mouse brain, support the hypothesis that ATM deficiency may lead to reduced developmental clearance of aneuploid neural cells [39]. Iourov et al. thus proposed that A-T is a disease of selective mosaic genomic instability, with neuronal aneuploidization in the A-T brain as a secondary genetic mechanism contributing to A-T brain pathology [21,24,40].

Iourov et al. also examined the rates of aneuploidy in AD [21]. AD is the most common cause of dementia. It is characterized by progressive cognition and memory deficits, with classic histopathological hallmarks of cortical atrophy, neuronal cell death, and stereotyped amyloid beta plaque and neurofibrillary tangle accumulation [41,42,43]. While general aneuploidy in the AD brain was not found to be significantly increased compared to controls, chromosome 21 aneuploidy (both monosomy and trisomy) was reported to be dramatically increased (10-fold), which lends support for the hypothesis that sporadic AD may be associated with trisomy 21 [21,35]. However, these data contrast with both FISH and scWGS studies, in which there was no observed selective gain of chromosome 21 in neurons of AD patients compared to controls [28,31,44]; technical differences including limited sampling could account for these differences.

Arendt et al., examined cortical neurons in AD patients and controls via slide-based cytometry and found DNA content was increased 2–3-fold in AD patients compared to controls. Furthermore, DCV was highest in regions of selective vulnerability, such as the entorhinal cortex, and corresponded with earlier age of death in AD patients (60–70 years old versus 80–90 years old) [45]. This phenomenon is also reported in controls, in which DCV decreases with age [30]. One hypothesis is that increased DCV contributes to higher disease burden, therefore resulting in an earlier age of death in AD patients. Increases in DCV in AD cortices as compared to cerebellum and controls have also been seen in PI flow cytometry and scWGS [31].

## 3. Copy Number Variation

### 3.1. Somatic Copy Number Variation in Health & Aging

**Figure 2:**
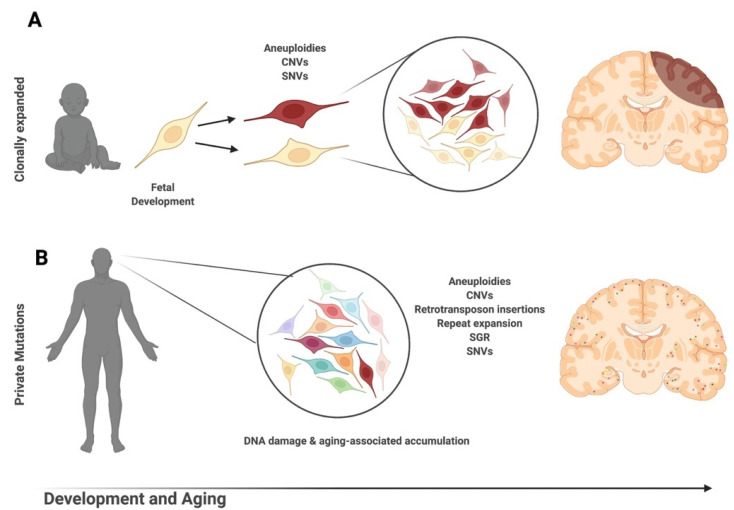
Clonal versus private somatic mutations in the human brain. (**A**) Somatic mutations that occur at different timepoints within embryogenesis or neurogenesis will result in clonal mutations shared by the brain and other body tissues or between brain cells of a single lineage. Clonal somatic aneuploidies, copy number variations (CNVs), and single-nucleotide variations (SNVs) have been described between the brain and peripheral lymphocytes (occurring early in embryogenesis), between the brain and other ectodermal-derived tissues (occurring after trilaminar disc formation), and between separate brain regions (occurring during neurogenesis). (**B**) Private or unique somatic mutations are unique to a single neural cell and accumulate throughout one’s lifespan. These somatic events may be independent of replication, as evidenced by their presence in post-mitotic cells. Aneuploidies, CNVs, retrotransposon insertions, repeat expansions, somatic gene recombination (SGR), and SNVs have all been described as occurring in post-mitotic neurons.

Somatic CNV occurs when the genome experiences gains and/or losses that change the number of copies of a particular gene amongst cells within the same individual. The presence of unique or shared CNVs across cells or tissues indicates that they likely arose within different stages of development (Figure 2). Similar to aneuploidies, the methodology used for detection can greatly alter the conclusions drawn about brain CNVs. Detected CNV size minimum and acceptable rates of variability in read depth may contribute to false calls and differences in reported CNV frequency [46,47]. With strict parameters, somatic CNVs > 1 Mb are estimated in ~9% of brain cells [46]. Stringent reanalysis of multiple single-cell datasets estimates that ~7% of neural genomes contain somatic CNVs ranging in size from 2.9 to 159.1 Mb with significant interindividual variability (0–24% of genomes containing somatic CNVs) [48]. Compared to non-neurons, neurons have more and larger CNVs that cover more territory within the genome [48,49].

Brain and regional CNVs and CNV rates can be identified through comparative analyses of brain regions and peripheral tissues. One method is array comparative genomic hybridization (CGH), where genomic DNA of experimental samples and references are labeled with different fluorochromes and hybridized onto an array of immobilized DNA targets. Differences in fluorescence intensities from a reference are used to identify genomic gains and/or losses [50,51]. An array-CGH study of CNVs > 50 kB from healthy subjects (including brain cortex, pons, and cerebellum) showed a single CNV in pons, but not the cerebellum or cortex within an individual [52]. Array-CGH analysis also revealed high heterogeneity of CNVs within tissues of a single individual. Of the 75% of CNVs that were CNS-specific (versus lymphocytes), 43% were unique to the frontal lobe and 32% to the cerebellum [53]. Across brain regions, deletions were more common than duplications in both young and aged adults [17,46,54]. However, the cerebellum was shown to have significantly larger deletions than the frontal lobe [53]. This is in agreement with the finding of increased DCV in the frontal lobe compared to the cerebellum [29], but the detected CNV gains [17,46,48,54,55] do not directly explain the estimated gain of ~250 Mb in the DNA content shown by Westra et al. [29]. As with aneuploidies, aged individuals have fewer neurons with CNVs, indicating that the presence of CNVs may affect the fitness of cells [48,56]. However, significantly more CNVs have been found in the aged brain compared to blood within the same individual [53], indicating that selective pressure against genomic mutations may still be less in the brain than in other tissues.

The genomic location of somatic CNVs provide clues about the mechanism of generation. CNVs and their boundaries are enriched in repetitive sequences (SINES/LINES, repeat elements, and noncoding RNAs) and telomeres [46]. Enrichment hotspots have been described in multiple studies, including potential enrichment within long genes such as *GPC6*, *NRNX3,* and *RBFOX1* [27,48].

### 3.2. Somatic Copy Number Variation in Neurodegenerative Disease

Increased copy number of neurodegenerative disease-related genes, even sparsely present within a brain, may have a significant impact on pathogenesis or heterogenous presentation of neurodegenerative diseases.

Parkinson’s disease (PD) and multiple system atrophy (MSA) are α-synucleinopathies [57]. PD is characterized by resting tremor, rigidity, bradykinesia, and other signs and symptoms associated with loss of dopaminergic neurons in the substantia nigra (SN) and other regions [58]. MSA is a rare and fatal disease characterized by combinations of parkinsonism, autonomic failure, ataxia, and pyramidal tract dysfunction associated with glial inclusions and cell loss [59]. In both familial and sporadic PD, copy number changes in *SNCA* have been described [60]. *SNCA* is located within a fragile genomic region that may be especially vulnerable to breaks during DNA replication [61]. This presents the intriguing question of whether somatic copy number changes may underlie sporadic disease that would be undetectable by standard lymphocyte-based genomic testing. One study of two individuals with early-onset PD using paired buccal and leukocyte samples demonstrated no *SNCA* gains in leukocytes, but a duplicate or triplicate copy number was found in 82% and 43% of oral mucosal cells [62]. The brain was not assessed in this study. Oral mucosa and brain tissue are of ectodermal origin as opposed to lymphocytes, which are of mesodermal origin, indicating that clonal, disease-relevant genomic changes in early development may be obscured when using blood as the predominant sample for genotyping.

Within dopaminergic neurons of the SN, there is evidence of somatic *SNCA* gains via FISH in PD patients [63]. While *SNCA* copy number increases were only observed in a small population of dopaminergic neurons (<1%), this could still be highly significant. SN dopaminergic neurons are significantly depleted in end-stage disease. Therefore, the ~1% of affected dopaminergic neurons may reflect only the surviving neurons within the region. The number of *SNCA* gains was negatively associated with onset age. Within this same study, the control case with the highest gains (1.87% of dopaminergic neurons) demonstrated incidental α-synuclein pathology with rare Lewy neurites. In a separate study, FISH analysis of cingulate cortex neurons demonstrated increased *SNCA* copy gains in MSA (2.8%) and PD (2.15%) compared to controls (1.12%) [64]. Furthermore, synuclein inclusions were more common in cells with CNVs than without in PD (22.1% vs. 5.7% neuromelanin positive) and in MSA (31.2% vs. 15.9% in olig2 positive). Whole-genome amplification and sequencing of two MSA cases in the same study found only one pontine neuron with a gain including the *SNCA* region [64]. Interestingly, one study in diseased brainstem and putamen (MSA) found non-neurons to have nearly all gains (>95%) compared to ~45% in neurons [64].

Demonstrating the very different results that may occur across techniques, array-CGH used to detect high level copy number changes of PD-relevant genes (*SNCA* included) detected no evidence of copy number changes [63]. A genome array study of frontal lobe in PD patients also demonstrated no specific gains or losses in *SNCA* but did detect CNVs unique to PD brains (versus controls) in other PD candidate genes (*BCL2, NRSN1,* and *RYR2*) [54].

In AD, there is also interest in the copy number of disease-related genes, especially *APP* (amyloid precursor protein)*,* since increased copies have been linked to Down syndrome-associated dementia [65]. In a study of 1511 whole brain exomes, a single AD patient was found with a triplication of the *APP* locus [66]. Increases in the *APP* copy number in AD cortical nuclei versus control using qPCR and PNA-FISH with *APP* probes have been reported, with no associated increase in chromosome 21 [31]. Therefore, it appears that a non-aneuploidy mechanism is driving *APP* CNVs in these neurons. However, targeted enrichment of AD-related genes in AD entorhinal cortex, with a sensitivity threshold of 10% of cells, found no CNVs of *APP*, *PSEN1*, *PSEN2*, or *MAPT* [67]. If increased copies are below 10% or involve partial *APP* sequences (<1 Mb), it may be undetectable using array or sequencing methods without ultra-high depth.

CNV changes have been further investigated in amyotrophic lateral sclerosis (ALS). ALS is a fatal neurodegenerative disease characterized by progressive muscle weakness because of the loss of spinal and cortical motor neurons [68]. Pamphlett et al. examined copy number differences between paired brain and blood samples and discovered 410 brain-specific CNVs (deletions > amplifications), with 121 found exclusively in ALS. Of these 121 ALS-specific CNVs, 24 were rare and overlapped in genic or promoter regions. Although no CNVs were identified in genes known to cause familial or sporadic ALS, CNVs were found in genes correlated with ALS pathogenesis, including *ATG7, GRIK1, GRIK2, FOXO3,* and *GGTLC2* [69]. In addition, three of these genes were previously noted in germline ALS copy number studies: *CSMD1, CNTN4,* and *GGTLC2* [70,71,72].

## 4. Retrotransposons

Retrotransposons compose ~40% of the human genome [73]. Part of a broader group of transposable elements, retrotransposons utilize a “copy and paste” mechanism to integrate into the genome via an RNA intermediate. This mechanism has allowed active retrotransposons to increase in copy number across the genome during evolution [74]. Retrotransposons are further categorized as either autonomous or non-autonomous retrotransposons. Autonomous retrotransposons are composed of long terminal repeat (LTR) retrotransposons and non-LTR-retrotransposons, so named because of the presence or absence of flanking LTRs. LTR-retrotransposons, such as human endogenous retroviruses (HERVs), comprise about 8% of the human genome [73,75]. Of the 31 HERV subfamilies, HERVk (HML2) is capable of unfixed endogenous retrovirus insertions [76]. Non-LTR retrotransposons, also known as Long Interspersed Nuclear Elements (LINEs), comprise about 17% of the human genome. LINE1 encoded proteins also mediate mobilization of Short Interspersed Nuclear Elements (SINEs), including Alu and SVA elements, by recognizing, binding, and integrating non-autonomous SINEs into the genome [77]. SINEs comprise 13% of the human genome [73]. Somatic reinsertion of retrotransposons can mediate functional changes through many mechanisms, including insertional mutagenesis, loss of function mutations, premature transcript termination, alternative splicing, chromatin alterations, gene silencing, promoter effects, changes in mRNA localization, processed pseudogene formation, and DNA damage [78,79,80,81,82,83,84,85].

Multiple endogenous defense mechanisms counteract the expression, function, and reinsertion of retrotransposons in the mammalian genome. Indeed, a minority of retrotransposons are expressed, and, of those, an even smaller minority appear to be functional because of mutations that eliminate open reading frames (ORFs) or produce inactivated translated proteins [86]. These defense mechanisms occur at both the transcriptional and post-transcriptional level: chromatin modification, DNA methylation, PIWI-interacting RNAs, RNA interference mechanisms, post-transcriptional degradation of the target TE transcript via siRNAs, and autophagy [87,88]. Of note, autophagy failure is implicated as the underlying disease pathology in multiple neurodegenerative diseases including ALS, PD, and AD (reviewed in: [89]).

### 4.1. Retrotransposons in the Normal Brain

Retrotransposons may play a role in normal brain physiology and development. However, more studies are required to verify functionality, especially in the human brain. LINE1 reactivation accompanies dopaminergic neuron maturation in post-mitotic somatic cell trans-differentiation models [90]. LINE1 inhibition also has been reported to impair this trans-differentiation potential. LINE1 reactivation and reinsertion may therefore create lineage-specific genomic mosaicism crucial to cell identity specification, providing support for the hypothesis that shared patterns of somatic mutations shape cellular identity [14]. LINE1 retroinsertion has also been implicated in early life and memory formation. Increased LINE1 copy number has been correlated with induced early-life stress [91]. Inhibition of LINE1 retrotransposition in the adult hippocampus impairs long-term memory formation in mouse models [92]. Furthermore, LINE1 copy numbers are increased in healthy human hippocampal neurons, although the exact prevalence is debated [93,94,95,96]. It has been speculated that retrotransposition may represent a form of neuronal plasticity, in which permanent genomic changes occur in response to experiences.

HERV expression is tightly regulated during development, and increased transcription of HERVk is detrimental for the development and function of cortical neurons in human-pluripotent-stem-cell-based systems [97]. LINE1 is also implicated as a heritable genetic contributor to somatic mosaicism via CNV formation. Rearrangements within inherited LINE1s can result in the deletion of proximal genomic regions, suggesting that LINE1-associated genomic regions are hotspots for somatic copy number variants in the brain [98]. LINE1 retrotransposition is normally tightly regulated through chromatin accessibility and transcription factors. Cell division promotes LINE1 retrotransposition because of the breakdown of the nuclear envelope, although it is not required [99,100]. The 5′UTR of LINE1 contains transcription factor binding sites for YY1, RUNX, and SRY (e.g., SOX2) families that mediate LINE1 expression [101,102,103]. SOX2 downregulation induces LINE1 expression [104]. Interestingly, SOX2 expression is decreased in developing, aging, and AD brains [105,106]. While LINE1 retrotransposition can occur in post-mitotic neurons and LINE1 expression is known to increase with age, the prevalence of LINE1 retrotransposition in the aged brain is unknown [107].

### 4.2. Somatic Retrotransposition in Neurodegenerative Disease

Somatic LINE1 retrotransposition has been indicated in several pathogenic processes, including neurodegenerative disorders, autoimmune disorders, and cancer [93,108,109,110,111]. In particular, LINE1 has been implicated in A-T, Rett syndrome (RTT), frontotemporal lobar degeneration (FTLD), ALS, and AD. These diseases can be divided into two categories: (1) diseases in which LINE1 expression/retrotransposition is increased because of mutations in genes that regulate retrotransposons and (2) diseases in which there is aging associated neurological degeneration and retrotransposon copy number/expression.

The first category includes A-T and RTT. LINE1 copy number is increased in A-T brains compared with healthy controls [112,113]. This increase is directly due to ATM deficiency, with *ATM* knock-out mice and *ATM*-deficient neural progenitor cells demonstrating significant increases in LINE1 retrotransposition [112]. Similar increases in LINE1 copy number have been demonstrated in RTT. RTT is a progressive neurodevelopmental disorder in which patients between 6–18 months of age develop severe impairments, including loss of speech and purposeful hand use, microcephaly, seizures, and autistic-like behaviors [114]. RTT is caused by mutation of the X-linked gene methyl CpG binding protein 2 (*MECP2*), leading to abnormal epigenetic regulation and LINE1 retrotransposition [109]. Somatic LINE1 insertions are significantly increased in both cortical neurons and non-brain tissues of RTT patients as compared to non-disease controls, with higher numbers of retrotranspositions in the brain compared to other tissues [113,115].

### 4.3. Aging-Associated Neurological Degeneration & Retrotransposon Copy Number/Expression

Aging-associated neurological degeneration and increased retrotransposon copy number/expression occur in FTLD, ALS, and AD. FTLD is the second most common cause of early onset (≤65 years) dementia and is marked by degeneration of the frontal and temporal lobes [116,117]. Forty percent of patients with FTLD have cytoplasmic inclusions of TDP-43, which normally functions in the repair of double strand breaks by non-homologous end joining [118]. TDP-43 cytoplasmic inclusions are also found in patients with ALS, AD, and other neurodegenerative disorders [119]. Transposable element transcripts are extensively bound by TDP-43, an association which is reduced in FTLD patients [120].

LINE1 has also been implicated in ALS. The first indication of retrotransposon activity in ALS patients came from the detection of increased levels of reverse transcriptase in non-brain compartments of ALS patients, at levels comparable to those found in human immunodeficiency virus (HIV)-infected patients [121,122]. The source and significance of this increased reverse transcriptase are still unknown. Tam et al. analyzed the transcriptomes of 148 ALS cortical tissue samples and identified three ALS transcriptomic clusters marked by (1) oxidative and proteotoxic stress (61%), (2) glial activation (19%), and (3) high levels of retrotransposon expression and signatures of *TARDBP*/TDP-43 dysfunction (20%) [123]. Loss of nuclear TDP-43 in neurons of ALS patients is also associated with decondensation of LINE retrotransposons and increased LINE1 DNA content [124]. HERVk has also been implicated in the pathophysiology of ALS, with HERVk *pol* transcripts increased in patients with ALS [125]. Sequencing of these ALS HERVk *pol* transcripts revealed several actively transcribed loci in the HML-2 and HML-3 subfamilies. In addition, HERVk reverse transcriptase (RT) protein was selectively expressed in neurons and was significantly increased in ALS patients compared to controls [125]. In order to examine whether HERVk expression could be causative for ALS, rather than correlative, Li et al. generated a transgenic mouse model overexpressing HERVk *env* in neurons. This mouse model developed progressive and specific loss of upper/lower motor neurons and showed double stranded DNA damage and nucleolar dysfunction, leading to a 50% mortality rate by 10 months [126]. Expression of HERVk in human neurons in vitro also caused toxicity, with decreases in cell number and neurite retraction [126]. However, further studies have presented conflicting data about whether HERVk transcripts are significantly elevated in ALS as compared to controls [126,127].

AD tau pathology is associated with increased retrotransposon expression, including HERVs and LINE1, in the human brain, as well as chromatin relaxation at selected HERV and LINE1 loci [128]. Mutant Tau *Drosophila melanogaster* also had significantly increased retrotransposon expression, indicating a causative effect of tau pathology on retrotransposon expression [128]. However, quantitative PCR of LINE1 revealed no difference in the LINE1 copy number in the brain and blood between AD patients and the aged-matched control group without dementia [129]. It remains unclear if the increased expression or reduced sequestration of retrotransposons in FTLD, ALS, or AD results in increased somatic insertion, genomic instability, or inflammation, thus contributing to disease progression. Therefore, further research on this topic is needed.

## 5. Somatic Repeat Expansion

Somatic repeat expansions are accumulations of short tandem repeat sequences (1–12 base pairs). These sequences can expand or contract over time through DNA polymerase slippage, non-allelic homologous recombination, or errors in DNA repair [130]. Several familial neurodegenerative diseases are caused by repeat expansions, including Huntington’s disease (HD), ALS, and spinocerebellar ataxia type 1 (SCA1); however, the presence of different repeat numbers across cells of a given individual may contribute to differential disease pathogenesis and phenotype.

HD is caused by an autosomal dominant CAG expansion in the Huntingtin gene (*HTT*). Between 50–70% of disease onset and severity is determined by the number of CAG repeats in *HTT* [131]. The CAG repeat is prone to expansion between successive generations leading to decreased age of onset and increased severity of disease, also known as genetic anticipation [132]. Although repeat expansion during early development may play a critical role in age of onset, instability during adulthood is key to disease progression. In HD, somatic expansion occurs most prominently in the brain compared to other body tissues, occurring preferentially in brain regions that are hardest hit by degeneration [133,134,135]. Sequencing of repeat length in grey matter indicates that neurons bear the brunt of repeat expansion [136,137]. Because neurons are post-mitotic, somatic repeat expansions are hypothesized to occur as a result of DNA damage rather than errors during DNA replication. The high energetic demands of neurons result in the production of high levels of reactive oxygen species, which endanger DNA integrity by chemically modifying bases and creating single and double stranded breaks [138]. Repeat sequences of DNA are prone to mutation because of the formation of secondary structures when the strands are separated either during replication, transcription, or DNA damage repair [139,140]. Genome-wide association studies (GWAS) identified DNA mismatch repair genes as significant modifiers of HD age of onset, in agreement with earlier work in mouse models [141,142,143,144]. Mechanisms of age-dependent CAG expansion have been demonstrated in neurons after terminal differentiation, demonstrating that somatic expansion does not require active cell division [145].

SCA1 is an autosomal dominant disorder caused by a CAG repeat in the *ATXN1* gene. SCA1 typically presents in the third to fourth decade of life and is characterized by progressive gait impairment, difficulties in speech and swallowing, and cerebellar atrophy [146]. As with HD, there is anticipation between generations, and expansion is thought to occur because of failure in DNA repair mechanisms [147]. A comprehensive quantitative analysis of CAG repeats across a range of CNS regions and peripheral tissues demonstrated similarly high levels of instability in cortex and neostriatum as those seen in HD. As opposed to HD, where these areas are correlated with disease severity, this pattern does not hold in SCA1 [148].

GGGGCC repeat expansions within the *C9ORF72* gene are present in ~35% of familial and ~6% of sporadic ALS and ~25% of familial and ~5% of sporadic FTLD, making it the most common known genetic cause of ALS and FTLD [149]. The pathogenicity of this hexanucleotide repeat has been attributed to both loss and gain-of-function mechanisms [150,151]. The presence of a large expansion may alter *C9ORF72* transcript expression because of mRNA quadruplex structures or epigenetic changes in accessibility produced by methylation. This can result in reduced C9orf72 protein, which plays important homeostatic roles in nucleocytoplasmic shuttling, endosomal transport, autophagy, and stress granules. Toxic gain-of-function mechanisms are implicated in both the bidirectionally transcribed mRNA and the translated protein products of *C9ORF72*. Repeat-containing sense or antisense RNA may cause cellular stress by sequestering essential RNA-binding proteins, therefore impeding RNA processing. C9orf72 dipeptide repeat proteins are synthesized from the sense and antisense transcripts through repeat associated non-ATG translation. Inclusions positive for dipeptide repeat proteins are found in CNS tissue from germline expansion-positive ALS and FTLD patients and are thought to disrupt nucleocytoplasmic transport and RNA processing [150,151].

Somatic expansion of the *C9ORF72* GGGGCC repeat has been found in different tissues of individuals with a germline repeat expansion in both ALS and FTLD [152,153,154]. One hypothesis for the development of phenotypically heterozygous ALS/FLTD is selective vulnerability of cortical or spinal cells to somatic expansion of the hexanucleotide repeat, even within individuals not harboring a germline expansion. In a study of ALS spinal cord in patients without an expansion found in blood, this hypothesis was not supported as no somatic expansion of the *C9ORF72* hexanucleotide was found [155]. Further studies are needed to support or disprove this hypothesis.

## 6. Somatic Gene Recombination

A novel form of somatic DNA changes in the brain, somatic gene recombination (SGR), was recently reported [156]. SGR was first described outside the CNS as somatic recombination of immunoglobulin genes, termed V(D)J recombination, in proliferating lymphocytes [157]. Bushman et al. reported increased amyloid precursor protein (*APP*) gene copy number in single neurons from SAD brains [31], leading to further examination of somatic changes affecting *APP* in AD. The presence of somatic, mosaic neuronal recombination and reinsertion of *APP* in the brain [156,158] could account for the copy number increases previously observed [31]. These recombined somatic copies were dubbed “gencDNAs,” so-called because the genomic intron-less sequences resembled complementary DNAs (cDNAs), thus the term genomic cDNAs (gencDNAs). *APP* gencDNAs were identified in frontal lobe neurons from both SAD and age-matched controls, including brain-specific splice variants (APP-751 and APP-695). In SAD, these *APP* gencDNAs were more numerous (~3–5-fold higher based on DNA in situ hybridization). Furthermore, long-read sequencing of *APP* gencDNA amplicons revealed greater sequence diversity in SAD brains, including frequent single base changes, insertions, and deletions. This included identification of 11 SNVs known to cause autosomal dominant familial forms of AD, but that were present in SAD neurons. The gencDNAs identified were often truncated *APP* species containing intra-exonic junctions (IEJs) where non-sequential exons are joined at non-canonical splice sites. IEJs have since been described in both DNA and RNA-based datasets, indicating potential transcription of these novel-spliced sequences [158,159]. Supporting the genomic presence of *APP* gencDNAs, novel insertion sites of the *APP* 5′ or 3′ untranslated regions into chromosomes 1, 2, 9, 10, and 12 were identified [158]. In addition, reads spanning *APP* exon-exon junctions with mate reads on chromosomes 1, 3, 5, 6, and 13 were also reported. An independent report documented *APP* gencDNAs in human AD hippocampus [160].

These findings are not without some controversy. Through a report using non-overlapping techniques, scWGS to 45× depth was unsuccessful in identifying gencDNAs in non-diseased and SAD neurons [161]. This study was limited to <10 neurons per brain and inconsistently identified germline pseudogenes within individuals, highlighting technical challenges that may impact gencDNA identification. The presence of a contaminated pull-down dataset was identified, highlighting the difficulty of library preparation and sequencing analysis without contamination [161]; however, repetition through 10 independent experiments that produced datasets devoid of contamination by two groups confirmed the original findings [158]. In addition, plasmid contamination could not explain the 10 genomic integration sites of *APP* sequences, nor the presence of 11 SNV familial mutations only in AD brain samples.

The creation of gencDNAs via SGR is hypothesized to require three main elements: gene transcription, reverse transcription of expressed RNA transcripts by an endogenous RT, and DNA strand breaks to allow for “retro-insertion” [156]. This proposed mechanism is supported by cell culture models of *APP* gencDNA formation in Chinese hamster ovary cells, a cell line with endogenous RT activity. Formation of gencDNAs required induction of DNA damage and was prevented with application of RT inhibitors [156]. The exact identity of endogenous RTs involved in *APP* SGR is unknown.

gencDNAs are reminiscent of germline processed pseudogenes that are classified as inactive evolutionary relics: they lack introns, show reduced sequence homology to the parent genes, are found in a non-wildtype gene location, and require similar components for reinsertion (gene transcription, RNA intermediates, RT activity, and DNA strand breaks). However, there are distinct and important differences. Processed pseudogenes are found at precise locations within the germline, which are stable during development and cell proliferation. In contrast, gencDNAs are somatic (not germline) and believed to occur in postmitotic neurons leading to diversity in location, number, and form amongst cells of the brain. “Somatic processed pseudogenes” have been described in proliferating cancer cells, contrasting with gencDNAs that occur in postmitotic cells. Additional similarities and differences between gencDNAs and processed pseudogenes have been reviewed [162].

Copy number changes of *APP* in the form of reinserted copies could be especially disease-relevant in AD. *APP* locus duplication causes autosomal dominant-early onset AD [163], and Down syndrome-associated triplication of the *APP* locus results in early onset dementia [65]. Additional copy number changes, in the form of gencDNAs, could result in SAD following a similar *APP* overexpressing disease process. It has been proposed that the protein products of variant *APP* sequences could result in more heterogenous biochemical processes than are utilized in classic Aβ peptide generation by γ- and β-secretases [164]. These varied products may escape therapeutic targeting by monoclonal antibodies raised against stereotyped Aβ peptide sequences, while secretase inhibitors might not be required for small products encoded by *APP* gencDNAs. Validation and clarification of SGR remain for future studies.

## 7. Somatic Single Nucleotide Variations

### 7.1. Somatic Single Nucleotide Variations in Development & Aging

The smallest and most common form of somatic genome changes affects single base pairs, SNVs. SNVs include transitions (purine to purine, pyrimidine to pyrimidine), transversions (purine ↔ pyrimidine), insertions, and deletions (the latter two are known as indels). SNVs in neurons accumulate because of endogenous and exogenous sources. Errors in DNA replication during development lead to shared SNVs in a cell’s downstream lineage. SNVs unique to single postmitotic cells can arise spontaneously secondary to DNA damage, such as through endogenous oxidative damage or exogenous factors such as radiation and smoking, or might arise through other processes such as SGR [158,165,166].

By birth, our brain cells already have accumulated several hundred clonal SNVs arising from proliferating progenitor cells [166]. During neurogenesis, ~5.1 SNVs accumulate per day per progenitor; this means that within a given cell division, ~8.6 SNVs occur per progenitor [167]. Three percent of SNVs found during development affect protein-coding sequences or gene regulation, and thus are likely to have functional consequences [167]. However, this same study found that SNVs are depleted in areas of open chromatin indicating efficient DNA repair in areas of active transcription. These clonal mutations are retained and can be detected into adulthood [168] without noticeable de-enrichment [169]. In adulthood, both clonal and private SNVs can be detected throughout the brain. In the adult prefrontal cortex, each neuron contains ~1500 somatic SNVs, with 60% of genomes containing at least one clonal SNV [168]. Compared to other tissues, the brain has a lower SNV rate than tissues with continual turn over or higher exposure to environmental mutagens (skin, lung, liver, small intestine, etc.) [170].

Age positively correlates with brain SNV load. When comparing average SNV prevalence per base pair in frontal lobe from those <10 years versus those >40 years, there was a 5.7-fold increase in SNVs [171]. In single neurons within the prefrontal cortex, an estimated 23 SNVs accumulate each year. By age 80, individual genomes of these neurons would contain approximately 2500 SNVs each [172]. The SNV rate in the brain does not appear to be uniform. Dentate gyrus neurons accumulated nearly two-fold higher (~40) SNVs per year [172]. Age was more strongly correlated with SNV load in basal ganglia, nucleus accumbens, hypothalamus, and hippocampus than the frontal lobe [170]. Together, this suggests that different regions of the brain are more vulnerable to SNV accumulation, which could stem from a number of factors (differential oxidative damage, DNA repair, exposure to environmental mutagens, or SGR processes). As with clonal SNVs detected during neurogenesis, there was no correlation between actively transcribed regions (by expression level or heterochromatin markers) and expected numbers of SNVs [170,173]. Open chromatin likely has enhanced DNA repair compared to more closed regions. It is notable that these estimates are based upon small sample sizes [168,172].

### 7.2. Somatic Single Nucleotide Variation in Neurodegenerative Disease

Many neurodegenerative disorders, including PD and AD, have a small minority of patients for which a single mutated allele causes autosomal dominant disease. The possibility that sporadic versions of these diseases, in which no germline mutant can be detected, could be caused by somatic mutations to risk genes is intriguing. Depending on when these mutations arise, they may escape detection in lymphocyte-based genotyping. One family has been described for a mosaic mutation causing inherited AD [174]. A *PSEN1* mutation (P436Q) was found in 8% of peripheral lymphocytes and 14% of cells in the cortex of an individual diagnosed with early-onset progressive parkinsonian syndrome with dementia. One child carried this mutation within the germline and had an even earlier presentation of progressive cerebellar syndrome with dementia, an indication that the parent mutation occurred before gastrulation [174]. A similar mosaic mutation resulting in inherited disease has been reported in ALS [175]. The index case demonstrated a *FUS* mutation at a low level of mosaicism difficult to detect in blood and saliva via whole exome sequencing, with higher allele fractions in hair. CNS tissue was unavailable for sequencing. The son of the index case had juvenile onset, and sequencing of blood demonstrated a heterozygous *FUS* mutation [175].

Brain whole exome sequencing is a common method used to interrogate somatic SNVs with a high likelihood of functional impact without a bias to particular genes. One such study sequenced exomes of matched hippocampus and blood samples from patients with SAD, vascular dementia, or no neurodegenerative disease [176]. While the vast majority of SNVs in SAD were detected in both the brain and blood (~97%), an average of 575 mutations were found in the brain only. No known pathogenic mutations were found in familial genes; however, non-pathogenic mutations were seen in the familial gene *PSEN1*, genes identified via GWAS (*BIN1*, *ABCA7*, and *PICALM*), as well as in genes related to SAD or Aβ processing [176]. A second study pairing brain and blood used laser capture to enrich for neurons within the hippocampal formation [160]. In SAD, the authors estimated that somatic SNVs within neurons increase at a rate of 0.53 per exome per year. When extrapolated to the genome, this would mean 22 SNVs per year within a whole genome [160]. This estimate is roughly half the ~40 SNVs per year predicted in isolated dentate gyrus [172], which may reflect differences in methodology. Finally, this study found one SAD patient with a pathogenic mutation in *PIN1* and 14 out of 52 SAD patients with at least one putatively pathogenic brain somatic mutation in pathways associated with tau phosphorylation [160]. Such low-level SNVs within the hippocampal formation may contribute to tau aggregation and propagation in SAD. When comparing SNV rates between disease and control groups, no difference was found in multiple studies [160,169,176].

One method to detect SNVs at low allele frequencies is to increase the sequencing depth via targeted enrichment of genes of interest. Genes must be selected a priori and are typically chosen for their direct connection to autosomal dominant disease or demonstration of increased risk in sporadic disease. One study examining *APP*, *PSEN1*, *PSEN2*, and *MAPT* in entorhinal cortex from 72 SAD and 58 non-AD controls found and validated three possibly damaging low-frequency SNVs: *MAPT* Q124K at a frequency of 1.1%, *PSEN2* S130L at a frequency of 1.6%, and *MAPT* S735A at a frequency of 0.7% [67]. A recent study targeting PD-associated genes in SN, frontal lobe, cerebellum, and blood from synucleinopathies and controls did not detect somatic SNVs in PD-associated genes [177]. Two studies using high-resolution melting curve analysis of *SNCA* amplicons demonstrated no evidence for low-level mosaicism in the coding regions of *SNCA* across multiple brain regions in PD, MSA, and Lewy body dementia (LBD), another α-synucleinopathy. This technique is limited to low-frequency SNVs and could miss high frequency SNVs that would occur early in embryogenesis [178,179]. A larger panel of 56 genes associated with neurodegenerative disorders (including AD and LBD) and 46 genes associated with cancer demonstrated no difference in SNV rates between brain regions or between gene panels in a cohort of SAD, LBD, and control patients. Seven SNVs were found in neurodegenerative disease genes (mean allele frequency 0.82%). Within a single individual, an SNV in neurodegenerative disease-associated gene *TAF15* was found across multiple brain regions at different frequencies (4.37–9.77%). The authors used a computational model of brain development to estimate that each individual has 10^5^–10^6^ pathologically mutated cells, and up to ~10% of all humans will have SNVs within neurodegenerative genes present diffusely across the brain [180].

Autosomal dominant AD is typically characterized by early disease onset (≤65 years) [181]. Thus, early-onset AD patients who are negative for germline SNVs are good candidates to screen for potential disease-causing somatic SNVs. Ultra-deep sequencing of eleven genes in brain from 445 early-onset SAD patients identified nine candidate SNVs at a frequency of between 0.22% to 10.8% [182]. No pathogenic mutations were found in familial genes, but mutations in other AD-related genes may have contributed to disease in these patients. One brain-specific mutation in *CD55* was later validated in a late-onset SAD patient in a study of AD-related genes in early- and late-onset SAD patients [183]. This mutation was present in the temporal cortex at an allele frequency of 0.4% and may contribute to AD pathogenesis via its role as a neuroprotective complement regulator [184].

RNA-seq has also been used to identify disease-causing mutations in AD brain. In a study examining multiple brain regions, 104 genes were found to have disease-causing SNVs in AD, converging on genes associated with the cytoskeleton, autism, and intellectual disability. SNVs in the autism-associated gene *ADNP* (activity-dependent neuroprotective protein) occurred more frequently in AD than in controls, with increased SNV frequency associated with increased tau burden [185]. The causal roles for identified SNVs in AD remain to be demonstrated.

## 8. Future Technologies, Research, & Therapeutics

Multiple technological challenges persist in detecting somatic variants, especially in post-mitotic populations such as neurons. Somatic variants, especially those that occur during aging, may occur at low frequencies, making their detection within whole genomes technologically and analytically difficult (reviewed in: [186,187]). While next generation sequencing (NGS) technologies have revolutionized genomics research, the error rates of NGS approaches (0.1–1%) remain a concerning problem [188]. Furthermore, DNA contamination, PCR induced error, misclassification, and DNA damage can lead to false positive reporting of mosaicism [189]. New sequencing and bioinformatics tools are actively being developed to help address these limitations and improve detection of low frequency events.

O2n-seq utilizes single-strand DNA circularization to create two different copies of one original molecule in a pair of paired-end reads [190]. If a variant is supported by only one DNA copy, an error must have occurred at the site and thus this sequence is discarded during subsequent data analysis. Only variants supported by both DNA copies are treated as true variants. This strategy may improve detection of de novo, low-frequency mutations on NGS platforms by eliminating sequencing errors, improving data efficiency, and reducing library bias seen with other methods. Other sequencing methods, such as duplex sequencing, Multiple Independent Primer PCR Sequencing, RePlow, and REBELseq, also promise to increase the detection of low-frequency events and reduce amplification bias/PCR-induced sequencing artifacts [191,192,193,194,195].

Detection of somatic variants is often accomplished through scWGS. Genome sequencing on a single-cell level requires massive amplification of genomes combined with a high sequencing depth. Often analysis is limited to small numbers of cells (<20 cells per sample) [168,172]. Techniques such as multiple displacement amplification (MDA) can provide sufficient amplification with a high-fidelity polymerase [47]. Sequencing typically requires at least 30X sequencing coverage. Bottleneck sequencing (BotSeqS) is an alternative to single-cell sequencing. Fragmented genomes are labeled with sequencing adapters and then diluted before PCR amplification to create a bottleneck allowing efficient random sampling of the genomic templates [171]. This method requires that the SNV is present on both the positive and negative DNA strands. Another methodology for expanding the genome to sufficient levels for sequencing is clonal expansion, where proliferating cells can be isolated and grown in culture [167]. This technique requires proliferating cells and thus cannot be applied to terminally differentiated, postmitotic neurons. However, it benefits from the use of cellular replication machinery, which has higher fidelity than ex vivo methods.

Analysis of larger sample numbers has been made possible through the use of RNA-sequencing and/or whole exome-sequencing datasets [169,170,173]. The Genome-Tissue Expression project has provided an excellent resource of matched RNA-seq and whole exome data from individuals. Successfully and accurately calling SNVs from RNA-seq data requires overcoming a high false discovery rate (representing mutations seen in RNA but not confirmed in matched whole-exome data) and a high false negative rate (representing mutations seen in whole-exome data not confirmed in RNA). A high false-positive rate can obscure true SNVs present at low allele-frequencies. It can be overcome by only considering genomic regions where both alleles are present in RNA-seq, using robust pipelines to remove artifacts and validating SNVs using whole-exome blood samples [170]. These analyses also limit detection to genes with sufficient expression but could provide a helpful guide to SNVs that may produce a functional impact within a cell.

Identifying novel insertion sites within human brain cells is an important component of validating somatic insertions into the genome. PCR methods, such as FLEA-PCR (flanking sequence exponential anchored-PCR) and pulldown enrichments, have provided insights into somatically acquired insertion sites in cancers [196,197]. However, as with other PCR-based methods, they are vulnerable to amplification and ligation artifacts. Furthermore, they are benefited by the clonal expansion characteristic of cancerous malignancy. A recent study leveraged long-read sequencing to identify somatic transposable element insertions in the *Drosophila melanogaster* head and midgut [198]. They applied Oxford Nanopore Technologies long-read sequencing to bulk genomic DNA from pooled midguts, which undergo clonal expansion, and heads from 60 individuals and sequenced to 85X coverage. Long-read sequencing allowed the identification of putative somatic integrations, as a single continuous sequencing read could fully contain the transposable element, insertion site, and validated target site duplication. This method of non-amplification-based long read sequencing analyzed only insertions supported by a single read (“singletons”) as potentially somatic. The frequency of transposition between gut and head samples could not be directly compared since somatic transposition in only a few cells of the head was below the level of detection, as compared to the clonally expanded midgut. The *Drosophila* head contains ~100,000 cells [199] and the haploid genome is ~180 Mbps [200]. By contrast, a single human brain has ~170 billion cells [201,202], with ~3100 Mbps average genome per cell (that may be significantly larger from cell-to-cell) [25,29,31].

These technologies further highlight the importance of crosstalk between fields within biomedical research. Many of these sequencing technologies were developed for the detection of novel, low-frequency somatic variants in cancer biology. Adapting these and other cancer technologies could provide increasingly fruitful results towards the understanding and treatment of sporadic neurodegenerative diseases. Cancer biology may also provide examples of genomic mosaicism understudied in the context of brain aging and neurodegeneration. Extrachromosomal circular DNA (ecDNA) has been characterized in tumor tissues [203]. These DNA species are excised from the linear genome, although they can potentially arise from any part of the genome as a consequence of DNA damage and repair. It is hypothesized that their origin is non-random and might arise from specific genomic hotspots [204]. Of interest, ecDNAs modulate gene copy numbers and transcription rates by different molecular means [205,206]. ecDNAs have also been found in somatically mosaic patterns in normal tissues, such as differentiated muscle and brain [204,207]. There is scant literature on human brain ecDNAs; however, several recent reviews have postulated that they may have a role in brain aging and neurodegeneration [208,209]. Technical and bioinformatics tools have been developed for genomic in-depth characterizations of ecDNAs [210]. These tools, combined with optimized wet lab approaches, may help to facilitate future investigations of ecDNAs within young, aged, and diseased brain.

Potential therapeutics for neurodegenerative diseases produced by somatic variants are already being investigated. Recent retrotransposon research in ALS and AD has led to multiple clinical trials on the safety and efficacy of HIV antiretroviral regimens for reducing clinical symptoms (NCT02868580, NCT02437110, NCT04500847, NCT04552795, NCT03706885). In addition, antisense oligonucleotide therapies could prove promising for modulating neurodegeneration impacted by somatic copy number variants or repeat expansion [211,212].

Identification of neural somatic variants, and therefore their targeted treatment, is hampered by our current genotyping strategies. Clinical identification of genetic variants relies on the identification of germline mutations in peripheral lymphocytes thus missing any brain-specific somatic variants. There is a critical need to identify alternative methods for identifying neural somatic mutations in patients. As previous studies have highlighted, because of the common embryonic origin of buccal and neural tissue, buccal swabs can provide insight into clonal, somatic variants that occur during embryogenesis. CNS-derived cerebrospinal fluid and blood exosomes are an additional peripheral source of neurodegenerative disease biomarkers and provide potential surrogate markers for neural somatic mutations [213]. Future imaging technologies may allow in vivo assessment of DCV towards classifying and possibly diagnosing disease states and progression. Collaborative multi-omic efforts, such as those orchestrated by the Allen Brain Institute and the Brain Initiative Cell Census Network, are promising avenues through which neural somatic variants can be identified. While tremendous advances have been made over the last two decades, further technological advances and collaborative efforts are important next steps towards understanding neural somatic variants and their role in multi-factorial neurodegenerative diseases.

## Abbreviations

AD—Alzheimer’s disease; ALS—myotrophic lateral sclerosis; A-T—Ataxia-telangiectasia; CGH—Comparative genomic hybridization; CNV—Copy number variation; DCV—DNA content variation; ecDNA—Extrachromosomal circular DNA; FTLD—Frontotemporal lobar degeneration; FISH—Fluorescence in situ hybridization; GWAS—Genome-wide association studies; HD—Huntington’s disease; HIV—Human immunodeficiency virus; IEJ—Intra-exonic junction; LBD—Lewy body dementia; LINE—Long interspersed nuclear element; LTR—long terminal repeat; MSA—Multiple system atrophy; PD—Parkinson’s disease; PI—propidium iodide; RT—Reverse transcriptase; RTT—Rett syndrome; SAD—Sporadic Alzheimer’s disease; scWGS—Single-cell whole genome sequencing; SGR—Somatic gene recombination; SINE—Short interspersed nuclear element; SNV—Single nucleotide variant.

## Appendix A

A comprehensive list of studies examining somatic variation in neurodegenerative diseased tissue can be found in Table A1.

**Table A1:** Summary of studies examining somatic mutations in neurodegenerative disease.

Disease	Somatic Variation	DNA Source	Technique	Somatic Finding	Reference
AD	DCV	16 SAD (7M:9F) & 16 ND (7M:9F); mean age 75 yo. 5 postmortem cortical regions	Slide-based cytometry	↑ DC in all AD cortical regions	Arendt et al., 2015 [45]
	CNV	32 SAD (14M:18F, 62–101 yo.) & 40 ND (15M:25F, 17–103 yo.) postmortem FR, CBLM	qPCR; PNA-FISH; flow cytometry	↑ DC & ↑*APP* copy number in SAD	Bushman et al., 2015 [31]
		20 SAD, 20 PD/LBD, & 14 ND; mean age 80–84 yo. 5 postmortem brain regions	Whole exome sequencing	1 *APP* gain in AD case	Keogh et al., 2018 [66]
		72 SAD (61–105 yo.) & 58 non-AD (18–97 yo.) postmortem ERC	Gene enrichment & amplicon sequencing	No CNVs in *APP, PSEN1, PSEN2, MAPT*	Sala Frigerio et al., 2015 [67]
	RTsp	422 SAD & 201 ND postmortem FR	RNA-seq	↑ LINE1 & HERVk expression	Guo et al., 2018 [128]
	SGR	7 SAD (1M:6F, 72–88 yo.) & 6 ND (3M:3F, 80–94 yo.) postmortem FR, CBLM	Amplicon sequencing; *APP* exonic pulldown; DISH	↑ *APP* gencDNAs in AD NeuN+ FR	Lee et al., 2018 [156]
		52 SAD (16M:36F, 70–96 yo.) & 11 ND (7M:4F, 57–89 yo.) paired blood & postmortem laser-captured HIF	Whole exome sequencing	APP IEJs in AD HPC	Park et al., 2019 [160]
	SNVs	2 related individuals (58 yo. F, 39 yo. F) blood & postmortem CTX	Allele-specific oligonucleotide hybridization	Mosaic *PSEN1* SNV in mother; germline heterozygous *PSEN1* SNV in child	Beck et al., 2004 [174]
		17 SAD (5M:12F), 2 VD (1M:1F), & 2 ND (2M) 46–94 yo. paired blood & postmortem HPC, CBLM	Whole exome sequencing	AD brain-specific SNVs in AD assoc. genes	Parcerisas et al., 2014 [176]
		72 SAD (61–105 yo.) & 58 non-AD (18–97 yo.) postmortem ERC	Gene enrichment & amplicon sequencing	2 *MAPT* & 1 *PSEN2* SNVs in AD & non-AD	Sala Frigerio et al., 2015 [67]
		372 EOAD (>66 years old at diagnosis), 73 LOAD, 1 FAD, & 52 ND blood & postmortem brain	smMIP assay & amplicon sequencing	9 candidate SNVs of benign/unknown sig.	Nicolas et al., 2018 [182]
		4 EOAD (59–68 yo.), 4 LOAD (79–89), 8 ND (53–88) blood & postmortem TC	Whole exome sequencing	1 brain-specific SNV in *CD55* in LOAD	Helgadottir et al., 2019 [183]
		20 SAD & 20 ND postmortem OB, HPC & 4 cortical regions	ddPCR; RNA-seq	Autism-assoc. *ADNP* SNVs in AD; 104 genes with disease-causing SNVs in AD	Ivashko-Pachima et al., 2019 [185]
		52 SAD (16M:36F, 70–96 yo.) & 11 ND (7M:4F, 57–89 yo.) paired blood & postmortem laser-captured HIF	Whole exome sequencing	1 *PIN1* pathogenic mutation in SAD	Park et al., 2019 [160]
ALS	CNV	32 SALS (22M:10F; 47–84 yo.) & ND (18M:6F) blood & postmortem brain	Microarray	24 CNVs in genic/promoter regions	Pamphlett et al., 2011 [69]
	RTsp	25 SALS, 3 FALS (mean 63 yo.) & 12 ND (mean 60 yo.) 4 CTX regions	RTqPCR	↑ HERVk pol expression in ALS	Douville et al., 2010 [125]
		148 SALS, 11 other neurologic disease, & 17 ND postmortem CTX	RNA-seq	↑ LINE1 expression in ALS	Tam et al., 2019 [123]
	RE	19 SALS (9M:7F:3unknown; 50–79 yo.), *C9ORF72* expansion negative; postmortem spinal cord	RepeatPrimer PCR & amplicon size genotyping	No somatic expansion of *C9ORF72* repeat	Ross et al., 2019 [155]
		ALS with or without *C9ORF72* repeat expansion blood, CNS, non-neural tissues	Southern blot	Intra-individual variation of *C9ORF72* repeat	Buchman et al., 2013 [152], Dols-Icardo et al., 2014 [153], Nordin et al., 2015 [154]
	SNV	2 related individuals (33 yo. M, 50 yo. F, living) blood & saliva	Whole exome sequencing	*FUS* mosaic SNV in mother; germline heterozygous *FUS* SNV in child	Hisahara et al., 2021 [175]
A-T	RTsp	7 A-T & 7 ND, 8–28 yo. laser capture of postmortem HPC	Taqman-based qPCR for ORF2 sequence	↑ LINE1 copy number in A-T	Coufal et al., 2011 [112]
		4 A-T, 2 RT, 72 other, & 20 ND postmortem neural & non-neural tissue	Whole-genome sequencing	↑ LINE1 copy number in A-T cortex	Jacob-Hirsch et al., 2018 [113]
FTLD	RTsp	FTLD & ND brain	Crosslinking-immunoprecipitation sequencing	↓ binding of RTsp by TDP-43 in FTLD brains	Li et al., 2012 [120]
	RE	FTLD with or without *C9ORF72* repeat expansion blood, CNS, non-neural tissues	Southern blot	Intra-individual variation of *C9ORF72* repeat	Buchman et al., 2013 [152], Dols-Icardo et al., 2014 [153], Nordin et al., 2015 [154]
HD	RE	3 HD (27–40 yo.) postmortem striatum	PCR amplification; small-pool PCR	↑ CAG repeats in striatal cells	Kennedy et al., 2003 [133]
		5 HD (3M:2F, 40–64 yo.) postmortem striatum & TC	PCR amplification & Southern blot	↑ CAG repeats in neurons vs. glia	Shelbourne et al., 2007 [136]
		24 HD young onset (20–41yo.) & 24 old onset (40–81 yo.) postmortem CTX & CBLM	Small-pool PCR	↑ repeat size assoc. with young onset	Swami et al., 2009 [134]
		7 adult-onset HD (2M:5F, 39–66 yo.) & 1 juvenile- onset HD (1M, 6 yo.), postmortem CNS & PNS tissue	Repeat length genotyping	↑ *ATXN1* CAG repeats in brain tissues	Mouro Pinto et al., 2020 [148]
MSA	CNV	5 MSA (55–76 yo.) & 30 ND (59–94 yo.) postmortem SN dopaminergic neurons	FISH	↑ *SNCA* copy number in MSA	Mokretar et al., 2018 [63]
		18 MSA (5M:13F, 52–82 yo.) & 17 ND (10M:7F, 59–92 yo.), postmortem cingulate CTX, CBLM	FISH; whole-genome sequencing	↑ *SNCA* copy number in MSA	Perez-Rodriguez et al., 2019 [64]
PD	CNV	8 PD (5M:3F, 63–81yo.) & 26 ND (18M:8F, 44–85yo.) postmortem FR	Microarray	CNVs detected in PD candidate genes (not *SNCA*)	Pamphlett et al., 2012 [54]
		2 PD living donors (40 yo. M, 23yo. M): mucosal cells, blood	FISH	↑ in 4p22.1 locus of *SNCA* in PD mucosa	Perandones et al., 2014 [62]
		41 PD (56–83 yo.) & 30 ND (59–92 yo.) postmortem SN dopaminergic neurons	FISH	↑ *SNCA* copy number in PD	Mokretar et al., 2018 [63]
		26 PD (20M:6F, 60–83 y.o) & 18 ND (10M:7F, 59–92 yo.) cingulate CTX & CBLM	FISH; whole-genome sequencing	↑ *SNCA* copy number in PD	Perez-Rodriguez et al., 2019 [64]
	SNV	28 PD (17M:11F, 62–90 yo.) postmortem SN & CBLM	HRM analysis of amplicons	No SNVs within *SNCA*	Proukakis et al., 2013 [178]
		511 idiopathic PD (age of onset 61 yo.) postmortem CBLM, SN, FR	HRM analysis of amplicons	No SNVs within *SNCA*	Proukakis et al., 2014 [179]
		20 PD/LBD (67–91 yo.) & 15 ND (64–97) blood & 4 postmortem brain regions	Gene enrichment panels	Brain-specific SNVs in neurodegenerative genes	Keogh et al., 2018 [180]
		25 sporadic PD (21M:4F, 55–88 yo.), 1 familial PD, & 12 ND (4M:8F, 69–104 yo.); postmortem SN	Gene enrichment panel; ddPCR	No disease-relevant SNVs detected	Leija-Salazar et al., 2020 [177]
RTT	RTsp	4 A-T, 2 RT, 72 other, & 20 ND postmortem neural & non-neural tissue	Whole-genome sequencing	↑ LINE1 copy number in RTT	Jacob-Hirsch et al., 2018 [113]
		5 RTT (5F, 17–21 yo.) & 5 ND (5F, 16–25 yo.) postmortem CNS & peripheral tissue	PCR-based targeted bulk sequencing	↑ LINE1 insertions in CTX neurons	Zhao et al., 2019 [115]
SCA1	RE	1 SCA1 postmortem CNS & peripheral tissue	Repeat length genotyping	↑ *ATXN1* CAG repeats in brain tissues	Mouro Pinto et al., 2020 [148]

AD = Alzheimer’s disease; ALS = amyotrophic lateral sclerosis; A-T = ataxia telangiectasia; CBLM = cerebellum; CNV = copy number variation; CNS = central nervous system; CTX = cortex; DCV = DNA content variation; ddPCR = digital lobe PCR; DISH = DNA in situ hybridization; EOAD = early-onset Alzheimer’s disease; ERC = entorhinal cortex; FALS = familial amyotrophic lateral sclerosis; FISH = fluorescent in situ hybridization; FR = frontal cortex; FTLD = frontotemporal lobar degeneration; HD = Huntington’s disease; HIF = hippocampal formation; HPC = hippocampus; HRM = high-resolution melting curve; IEJs = intra-exonic junctions; LBD = Lewy Body Dementia; LOAD = late-onset Alzheimer’s disease; MSA = multiple system atrophy; ND = non-diseased; OB = olfactory bulb; PD = Parkinson’s disease; PNA-FISH = peptide nucleic acid fluorescence in situ hybridization; PNS = peripheral nervous system; RE = repeat expansion; RTqPCR = quantitative reverse transcription PCR; RTsp = retrotransposons; RTT = Rett’s syndrome; SALS = sporadic amyotrophic lateral sclerosis; SCA1 = spinocerebellar ataxia type 1; SGR = somatic gene recombination; smMIP = single-molecule molecular inversion probes; SNV = single-nucleotide variation; TC = temporal cortex; VD = vascular dementia; yo = age at time of death (years old).
